# Predict multi-type drug–drug interactions in cold start scenario

**DOI:** 10.1186/s12859-022-04610-4

**Published:** 2022-02-16

**Authors:** Zun Liu, Xing-Nan Wang, Hui Yu, Jian-Yu Shi, Wen-Min Dong

**Affiliations:** 1 School of Computer Science, Northwestern Polytechnical University, Xi’an, 710072 China; 2 School of Life Sciences, Northwestern Polytechnical University, Xi’an, 710072 China

**Keywords:** Machine learning, Drug–drug interactions, Multi-type interactions, Prediction, Cold start

## Abstract

**Background:**

Prediction of drug–drug interactions (DDIs) can reveal potential adverse pharmacological reactions between drugs in co-medication. Various methods have been proposed to address this issue. Most of them focus on the traditional link prediction between drugs, however, they ignore the cold-start scenario, which requires the prediction between known drugs having approved DDIs and new drugs having no DDI. Moreover, they're restricted to infer whether DDIs occur, but are not able to deduce diverse DDI types, which are important in clinics.

**Results:**

In this paper, we propose a cold start prediction model for both single-type and multiple-type drug–drug interactions, referred to as CSMDDI. CSMDDI predict not only whether two drugs trigger pharmacological reactions but also what reaction types they induce in the cold start scenario. We implement several embedding methods in CSMDDI, including SVD, GAE, TransE, RESCAL and compare it with the state-of-the-art multi-type DDI prediction method DeepDDI and DDIMDL to verify the performance. The comparison shows that CSMDDI achieves a good performance of DDI prediction in the case of both the occurrence prediction and the multi-type reaction prediction in cold start scenario.

**Conclusions:**

Our approach is able to predict not only conventional binary DDIs but also what reaction types they induce in the cold start scenario. More importantly, it learns a mapping function who can bridge the drugs attributes to their network embeddings to predict DDIs. The main contribution of CSMDDI contains the development of a generalized framework to predict the single-type and multi-type of DDIs in the cold start scenario, as well as the implementations of several embedding models for both single-type and multi-type of DDIs. The dataset and source code can be accessed at https://github.com/itsosy/csmddi.

**Supplementary Information:**

The online version contains supplementary material available at 10.1186/s12859-022-04610-4.

## Introduction

Drug–Drug Interactions (DDIs) are pharmacological reactions occurring in human bodies when two or more different drugs are taken together. These reactions may be beneficial or harmful to patients. According to the report released by the US Centers for Disease Control and Prevention in 2010, about 10% of patients in American is under taking five or more medications [1], nearly twice as many as in 2000. During the same period, the number of people taking at least two drugs increased from one quarter to one third of the population. Similar statistics are also reported throughout Europe [1]. However, the fact that many DDIs cannot be identified during clinical trial phases, increases the risk of adverse drug reactions (ADRs). A report from the American Academy of Medicine states shows that about 44,000 to 98,000 people died each year due to medication errors. Among the death cases, about 7000 deaths are caused by ADRs [2], which have become the fourth leading cause of death in the US behind heart disease, cancer and strokes [3]. Therefore, it is extremely important to predict unknown DDIs before medications are approved or administered. However, it is a high cost to detect DDIs by biological or pharmacological assays. DDIs prediction by computational methods can assist in identifying potential DDIs during clinical trials [4].

In terms of the pharmacological need, DDIs prediction can be divided into single-type prediction and multi-type prediction. The former task can be seen as a binary classification, which predicts whether an interaction between two drugs or not. So far, many DDIs prediction methods focus on this type of task [5–8]. The task of multi-type DDIs prediction can be seen as a multi-class classification, which not only predicts whether an interaction between two drugs exists or not, but also outputs its specific type. For this task, several methods, especially deep learning-based methods, have been exhibit their potentials. Ryu et al. [9] and Ma et al. [10] use deep neural networks and graph autoencoder models to perform the multi-type DDIs prediction respectively. Zitnik et al. [11] construct a heterogeneous network integrating DDIs and Drug–Protein Interactions (DPIs), then use graph convolutional neural networks to learn the embedding representation of drugs to predict interaction types. Lin et al. [12] propose a MDF-SA-DDI model, which is based on the multi-source drug fusion, multi-source feature fusion and transformer self-attention mechanism to predict the potential multi-type DDIs.

**Fig. 1:**
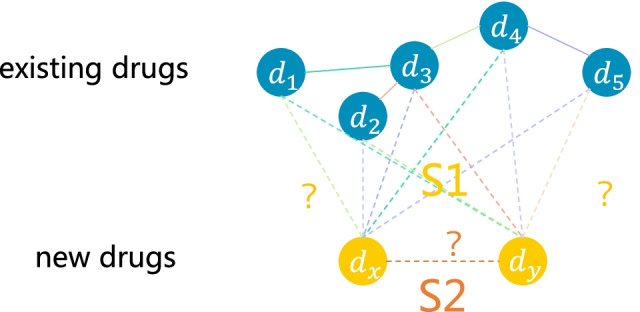
DDIs prediction in cold start $S1$ and $S2$*.* Blue nodes represent the existing drugs, and the lines between drugs represent their interactions, in which different colors represent different link types. The drugs above (in blue) are existing drugs in the DDIs network, and the one below (in yellow) are the new drugs. The cold start problem $S1$ refers to the prediction of interactions between the new drugs and the existed drugs, while $S2$ refers to the prediction of the interaction between two new drugs

The above-mentioned methods only pay attention to the scenario that requires to infer new interactions between drugs having approved interactions. Since DDIs are usually organized into a network [13], the scenario can be regarded as the ordinary link prediction in network science. Thus, drugs can be represented as vectors by embedding the DDI network and closed drugs in the embedded space are considered as interaction pairs. However, these methods cannot handle the cold start scenario that requires to predict interactions for new drugs not in the DDI network. Cold-start DDI prediction falls into two categories: $S1$ and $S2$*.* [14, 15]. $S1$ predicts interactions between new drugs and the drugs in the network while $S2$ predicts interactions between new drugs. A toy example is shown in Fig. 1.

The main obstacles of cold start DDI prediction are generating the embeddings of new drugs. Compared with the ordinary DDI prediction, the cold-start DDI prediction faces the difficulty that new drugs have no connections with the existing drugs in the DDI network. Therefore, we cannot utilize the network topology to generate embedding representations for new drugs. Some researchers are currently attempt to address this issue by bridging drug chemical or pharmacological properties to drug embeddings. For example, Shi et al. [14] use SVD method to get the embedding representation of drugs, then conduct the single-type DDI predictions in the cold start conditions $S1$ and $S2$. Celebi et al. [15] evaluate the performance of several knowledge graph embedding methods, including TransE, for single-type DDIs prediction in $S1$ and $S2$. Yu et al. [16] use a non-negative matrix factorization method to predicate the single-type DDIs in $S1$.

In this paper, we propose a computing model for single-type and multi-type DDI predictions in cold start scenario, named as CSMDDI. In this model, we use RESCAL-based method to get the embedding representations of drugs and DDI types and then learning a mapping function to bridge the drugs attributes to their embeddings to predict DDIs. The main contribution of CSMDDI contains the development of a generalized framework to predict the single-type and multi-type of DDIs in the cold start scenario, as well as the implementations of several embedding models for both single-type and multi-type of DDIs.

The rest of paper is organized as follows. Section 2 introduces the data selection and statistics. Section 3 presents the overall procedure of CSMDDI, including embedding representation learning, mapping function learning and predictors. Section 4 validates the performance of CSMDDI. Section 5 concludes our findings.

## Data

**Fig. 2:**
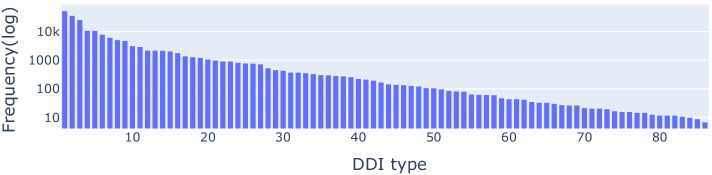
Frequency distribution of DDI types

The data used in this paper is extracted from DrugBank database (Version 5.1). DrugBank states DDI entries by sentences. For example, the interaction between Bivalirudin (DrugBank ID, DB00006) and Aceclofenac (DrugBank ID, DB06736) is given by “Aceclofenac may increase the anticoagulant activities of Bivalirudin”. We can manually identify a tuple (Aceclofenac, Bivalirudin, the anticoagulant activities, increase) in form of (drug1, drug2, mechanism, action). The “mechanism” and “action” are combined as a reaction type, for example “the anticoagulant activities increase” is the type of DDI between the drug pair (Aceclofenac, Bivalirudin). In order to extract all the reaction types from these sentences in DrugBank, we use StanfordNLP tool [17] to do this extraction. Detailed extraction procedures can be found in reference [18]. After extraction, we remove the drugs that react with less than 20 drugs to relieve the impact of too many reaction types on model predictions. And the final frequency distribution of all types is shown in Fig. 2 (all types are sorted by quantity). It can be seen that in Fig. 2, the imbalance of all types is clearly significant and the first five types account for large proportions in the whole space. In addition, we collected a set of binding proteins of each drug, including carriers, transporters, enzymes and targets (shorted as CTET) [19, 20]. After removing some proteins who have no interactions with the all drugs, there are total 1493 kinds of CTET proteins remaindering. We use the 1493 CTET proteins to code each drug into a fixed-length binary feature vector. Namely if drug ${d}_{i}$ binds protein ${p}_{j}$ in CTET, then the $j$-th element in ${d}_{i}$’s feature vector is 1, otherwise is 0. The vector example of drug’s binding with CTET protein is shown in Table 1.

**Table 1:** Drug attribute feature: CTET

	Polypeptides					
	*P*_1_	*P*_2_	*P*_3_	…	*P*_1492_	*P*_1493_
Universal Protein Resource identifier	P02768	O15540	P02753	…	Q8NEC5	P00480
$Dru{g}_{i}$	1	0	1	…	1	0

Finally, the summary of the collected data is as follows: the DDI network contains 1317 drugs, which are represented as 1493-dimensional binary indicating vectors of binding CTET proteins, and 198,697 DDIs, which are grouped into 86 pharmacological types (Additional file 1).

## Method

**Fig. 3:**
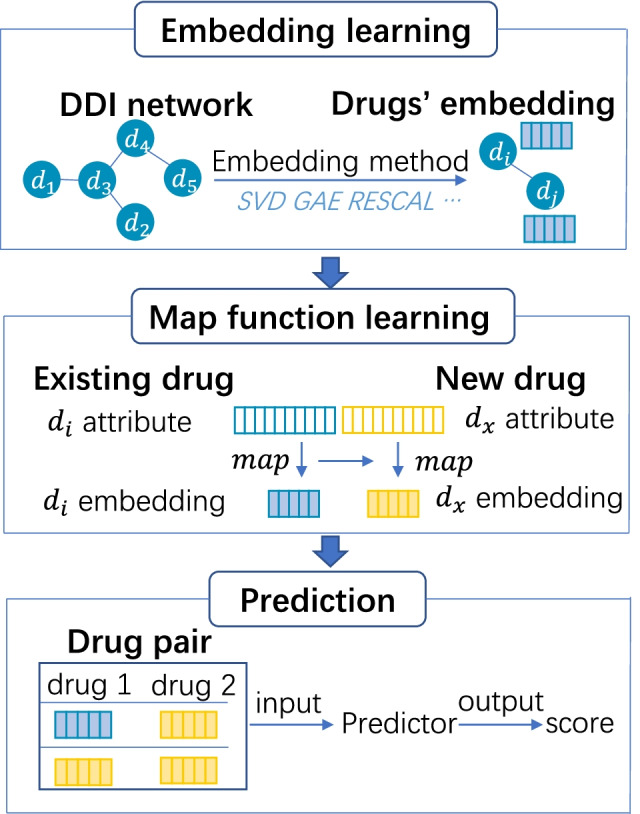
Computing framework of CSMDDI model. Embedding learning will learn the embeddings of the all existing drugs and DDI types. Mapping function learning will learn a mapping function between DDI network topology and drug’s attribute. Prediction will use the learned embedding vectors of a drug pair (one is existing drug and another is new drug, or two of them are new drugs) as the input, and then choose a predictor to output their prediction score. A higher score indicates a higher probability of this interaction occurrence

The computing framework of CSMDDI model contains three steps which are detailed in Fig. 3. The first step is to learn the embedding representations of drugs (existing drugs) in the DDI network. Several state-of-the-art embedding methods are accommodated, including SVD [14], GAE [21], TransE [22] and RESCAL [23]. In this step, each drug in the DDIs network is embedded as a vector. The second step is to learn a mapping function, which bridges drugs’ attributes to their embedding representations and is generated from existing drugs. Sequentially, we apply the mapping function on the attributes of new drugs, which are not in the network, to obtain their embedding representations. This step is the core to handle the cold start scenario. The third step is to predict single-type and multi-type interactions between new drugs and existing drugs. Taking the embedding vectors of an existing drug and a new drug as the input, the prediction score is output by the predictor, a higher score indicates a higher probability of the interaction occurrence in this drug pair.

### Problem formulation

Here we briefly explain the notation to be used in the following sections and give the goal of DDI predication. Giving an undirected DDI network, in which nodes represent drugs and edges represent interactions. Suppose there are *n* drugs, represented as *D* = {*d*_1_, *d*_2_, …, *d*_*n*_}. The adjacency matrix of the DDI network is represented by ***A*** = *a*_*ij*_ ∈ ${{\varvec{R}}}^{n\times n}$, where *a*_*ij*_ = 1 if there is an interaction between drug *d*_*i*_ and drug *d*_*j*_, otherwise *a*_*ij*_ = 0.

Drug attributes used in this paper are the drug binding proteins. Each drug can be represented as a CTET binary vector ***F***_*i*_ = {*f*_*i*1_, *f*_*i*2_, …, *f*_*ip*_} and all the drugs in the network are characterized as a matrix ***F*** ∈ ${{\varvec{R}}}^{n\times p}$. Let the embedding vector of drug *d*_*i*_ be ***E***_*i*_ = {*e*_*i*1_, *e*_*i*2_, …, *e*_*id*_}, where *d* is the dimension of learned embedding vectors, and the matrix ***E***∈${{\varvec{R}}}^{n\times d}$ be the embedding matrix of the all drugs. The representation of new drugs is same as the existing drugs, excluding their subscript is *x* or *y* (see Fig. 1).

The goal of CSMDDI model is to determine the DDI occurrence and their interaction types between existing drug *d*_*i*_ and new drug *d*_*x*_ or between two new drugs *d*_*x*_ and *d*_*y*_.

### Embedding learning

**Fig. 4:**
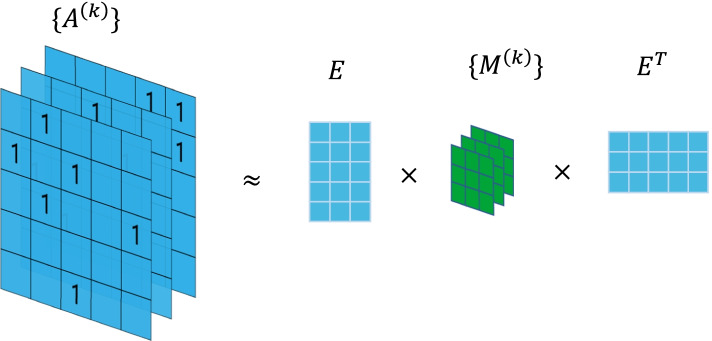
Factorization of adjacency matrices in RESCAL

The result of DDI predictions depends highly on the representations learned by the embedding learning part (the first part of Fig. 3). Therefore, it is important to select a good network embedding method. In order to get the embedding of drugs and interaction types simultaneously, we choose RESCAL model as the baseline embedding method to learn their representation. RESCAL [23] is a bilinear semantic matching model in relational learning. Suppose there are *n* nodes and *m* relationships in a relational network, adjacency matrix ***A***^(*k*)^ ∈ ***R***^*n*×*n*^, *k* = *{*1, 2, …, *m}* represents *k*-th relationship in the network. If node *i* and node *j* have a connection in the *k*-th relationship, then its element ${a}_{ij}^{(k)}=1$, otherwise ${a}_{ij}^{(k)}=0$. All adjacency matrices of these *m* relationships can be stacked up to the third dimension to form a three-dimensional adjacency tensor, as shown in Fig. 4. From the perspective of decomposition, the three-dimensional adjacency tensor can be approximately decomposed into an embedding representation ***E*** of nodes and the relation ***M***^(*k*)^ in the *k*-th relationship. The decomposition formula is

1 $$\widehat{{{\varvec{A}}}^{\left({\varvec{k}}\right)}}={\varvec{E}}{{\varvec{M}}}^{\left({\varvec{k}}\right)}{{\varvec{E}}}^{{\varvec{T}}}.$$

According to Eq. (1), for single-type DDI prediction, we can only learn one embedding matric ${\varvec{M}}$ which models the DDI between two drugs. As for multi-type DDI prediction, we model each DDI type as one embedding matric ${{\varvec{M}}}^{\left({\varvec{k}}\right)}$. The training loss of RESCAL is defined as

2 $$l={\sum }_{{\varvec{k}}}{\Vert {{\varvec{A}}}^{\left({\varvec{k}}\right)}-{\varvec{E}}{{\varvec{M}}}^{\left({\varvec{k}}\right)}{{\varvec{E}}}^{{\varvec{T}}}\Vert }_{2}^{2}$$

### Mapping function learning

The above embedding learning part can produce the embedding representations of the existing drugs in the DDI network. But embeddings of new drugs cannot be directly acquired, because they are not in the DDI network. Here, we provide a straightforward approach is to learn a mapping function which can bridge drug’s attributes to its topology embedding. Due to we have get the existing drugs topology embedding and also known the attributes of these drugs, here, in order to reduce the complexity, we choose a linear function to learn their mapping. The linear mapping is used in this work and defined as

3 $${\varvec{FB}} = {\varvec{E}},$$

where ***B*** is the mapping coefficient, ***F*** is the attribute matrix of drugs, and ***E*** is their embedding representations. Considering that the attributes of drugs are possibly colinear, we utilize partial least squares regression to learn the mapping function.

To obtain the mapping coefficient ***B***, we need to map ***F*** and ***E*** to their respective potential spaces [24]:

4 $${\varvec{F}} = {\varvec{TP}}^{T} + error_{1} ,$$

5 $${\varvec{E}} = {\varvec{UQ}}^{T} + error_{{2}} ,$$

where ***T*** = ***FX*** is the projection of ***F*** in the ***X*** direction, ***U*** = ***EY*** is the projection of ***E*** in the ***Y*** direction, ***P*** and ***Q*** are the loading matrices of ***F*** and ***E*** respectively. Finally, the mapping coefficient ***B*** = ***XY***^*T*^.

### Predictor

After getting the embedding representations of new drugs and existing drugs, we can predict their potential interactions. In the embedding learning part, we have selected RESCAL as the baseline embedding method. Therefore, we define the predictor as a score function between drug *d*_*i*_ and drug *d*_*j*_ in the *k*-th type of DDIs as

6 $$score = {\varvec{E}}_{i} {\mathbf{M}}^{(k)} {\varvec{E}}_{j}^{T} .$$

where ***E***_*i*_ and ***E***_*j*_ is the embedding representation of drug *d*_*i*_ and *d*_*j*_ respectively.

However, have mentioned above, in the CSMDDI model, the embedding learning method can be replaced by other embedding approaches, such as SVD, GAE and TransE etc., the corresponding predictor should also be changed to fit the prediction. For example, if we select SVD and GAE as the embedding method, their score function can be defined as

7 $$score = \left\langle {{\varvec{E}}_{i} ,{\varvec{E}}_{j} } \right\rangle = {\varvec{E}}_{i} {\varvec{E}}_{j}^{T} .$$

where < , > is the inner product of **E**_*i*_ and **E**_*j*_, *i* ≠ *j*.

If we select TransE as an embedding model, we can train a random forest classifier [19] as the predictor and the final DDIs prediction score of the two drugs is the output of the random forest classifier.

## Experiments

### Experimental setting

In our experiments, we use tenfold cross-validation (CV) to evaluate the predictive performance in the case of single-type and multi-type DDI. In the 10-CV, all drugs are equally split into 10 groups, of which only one group is left as the set of new drugs for testing to ensure the predication is in a cold start scenario. The other 9 groups are as the existing drugs constructed the DDI network in training phase.

The evaluation metrics are AUC (area under the receiver operating characteristic curve) AUPR (area under precision–recall curve) and F1-score. Particularly, the values of AUC and AUPR in the case of multi-type DDI prediction are averaged by all the DDI types because they are originally designed for binary classification. Additionally, F1-micro metrics is selected in the multi-type DDI prediction due to it is suitable for evaluating multi-classification. Moreover, when tuning model parameters, we set the dimension of drug embedding vector to 200 for all the models and determine other parameters (e.g., optimal learning rate, penalty term coefficients) by Grid Search. Specific scopes in grid search are as follows: both learning rate and penalty term coefficient are in {0.001, 0.01, 0.1}. We set the maximum iteration number as 1000 and stop the iterations early when there occur 3 loss values greater than the optimal loss value.

The evaluation metrics are both AUC (area under the receiver operating characteristic curve) and AUPR (area under precision–recall curve). Particularly, the values of AUC and AUPR in the case of multi-type DDI prediction are averaged by all the DDI types because they are originally designed for binary classification. AUPR is more appropriate than AUC in the case of imbalance classes. Moreover, when tuning model parameters, we set the dimension of drug embedding vector to 200 for all the models and determine other parameters (e.g., optimal learning rate, penalty term coefficients) by Grid Search. Specific scopes in grid search are as follows: both learning rate and penalty term coefficient are in {0.001, 0.01, 0.1}. We set the maximum iteration number as 1000 and stop the iterations early when there occur 3 loss values greater than the optimal loss value.

### Baselines

In CSMDDI model, we use RESCAL method as the baseline embedding to learn the representation of drugs. It can be altered by relative embedding method such as SVD [14], GAE [21], TransE [22] and so on. We compared the embedding method in the framework of CSMDDI in single-type prediction of DDIs in the cold start scenarios $S1$ and $S2.$ We also use the state-of-the-art DDI prediction method DeepDDI [9] and DDIMDL (Deng et al., 2020) as the baseline to verify the performance of CSMDDI. The comparison baselines descript as follows.

- **SVD** Singular value decomposition (SVD) [14] is a commonly used method of matrix factorization. It can be applied to reduce the dimensions of samples or calculate embedding representation. Its predictor score function of prediction is Eq. (7).
- **GAE** Graph AutoEncoder (GAE) [21] is a graph neural network model which includes an encoder and a decoder. The encoder is a multilayer graph convolutional network (GCN) [25] and the predictor score function also is Eq. (7).
- **TransE** TransE [22] is a typical representative distance-based model in knowledge graph embedding. It represents entities and relationships in the knowledge graph as vectors in the same space. As for its predictor, we use a random forest classifier. TransE + RandomForest is used for comparison both in single-type and multi-type DDI prediction.
- **DeepDDI** [9] is one of the earliest multi-type DDI prediction model, which uses the chemical substructure similarity of the drugs as the input and predicts the interaction type through a DNN. DeepDDI is used for comparison both in single-type and multi-type DDI prediction.
- **DDIMDL** [18] constructs similarity matrices based on different drug features and adopts a DNN predictor to perform DDI prediction for each feature. DDIMDL is used for comparison both in single-type and multi-type DDI prediction.

### Result

#### Prediction of single-type DDIs

The comparison results of single-type DDIs prediction are shown in Table 2 and the suffix of CSMDDI represents different embedding method applied in CSMDDI, highlighted in bold indicate the best perfomance. Here, since the training of TransE requires negative sample sampling, we follow a popular negative sampling strategy [26], which generates negative samples by randomly replace the tail in the DDI triple according to a uniform distribution and keeps the number of negative samples equal to the number of positive samples. From Table 2, it can be found that CSMDDI-RESCAL achieves the best performance compared with CSMDDI-SVD, CSMDDI-GAE and TransE + RandomForest in both $S1$ and $S2.$

**Table 2:** The prediction results of single-type DDIs

Methods	Single-type DDIs					
	*S*1			*S*2		
	AUC	AUPR	F1	AUC	AUPR	F1
CSMDDI-SVD	0.8137	0.6121	0.3735	0.7266	0.4783	0.3698
CSMDDI-GAE	0.7223	0.4435	0.3735	0.6387	0.3390	0.3726
CSMDDI-RESCAL	**0.8861**	**0.7270**	**0.4065**	**0.7709**	**0.5270**	**0.3751**
TransE + RandomForest	0.6451	0.4371	0.3730	0.4391	0.1116	0.3679
DeepDDI^(S)^	0.7267	0.5087	0.3725	0.7254	0.4804	0.3745
DDIMDL^(SP)^	0.7556	0.5592	0.3732	0.7215	0.4729	0.3718

(S) denotes that the inputs of the prediction method are chemical substructures

(P) denotes that the inputs of the prediction method are drug-associated proteins

It can also be seen that CSMDDI-RESCAL achieves the best performance compared with DeepDDI and DDIMDL. Additionally, the results indicate that the values of both AUC, AUPR and F1 score in the task of $S2$ are generally lower than those of $S1$. The reason is as follows. Each testing drug pair in task $S1$ only contains one new drug, while each testing drug pair in task $S2$ contains two new drugs. Due to the new drugs embedding come from the output of mapping function, it surely contains more errors in task $S2$, leading to its performance is lower than task $S1.$

#### Prediction of multi-type DDIs

Due to SVD and GAE are not fitted to be applied in multi-type DDIs prediction, we only compare CSMDDI with TransE + RandomForest, DeepDDI and DDIMDL. Table 3 shows the results of the prediction, the best are highlighted in bold. In Table 3, both AUPR and F1 score of CSMDDI-RESCAL achieves excellent performance in $S1$. For AUPR, it improves more than 12% compared with others, and more than 8% in F1-micro metrics. However, AUC of CSMDDI-RESCAL is lower than DeepDDI and DDIMDL, the reason is that DeepDDI and DDIMDL correctly predict more negative samples while CSMDDI-RESCAL correctly predict more positive example, leading to AUPR and F1 score of CSMDDI-RESCAL in $S1$ is superior to others but AUC is lower.

**Table 3:** The prediction result of multi-type DDIs

Method	Multi-type					
	$S1$			$S2$		
	AUC	AUPR	F1-micro	AUC	AUPR	F1-micro
TransE + RandomForest	0.8163	0.2853	0.5163	0.3843	0.0926	0.4040
DeepDDI^(S)^	**0.8864**	0.4638	0.6042	**0.4500**	0.1720	0.4825
DDIMDL^(SP)^	0.8832	0.4597	0.6292	0.4337	**0.2062**	**0.5173**
CSMDDI-RESCAL	0.8658	**0.5865**	**0.7168**	0.4313	0.2003	0.5103

(S) denotes that the inputs of the prediction method are chemical substructures

(P) denotes that the inputs of the prediction method are drug-associated proteins

Generally, in multi-class prediction, AUPR is more appropriate than AUC in the case of imbalance classes. From Table 3, we can also see that the AUC, AUPR and F1 score of CSMDDI-RESCAL in $S2$ is slightly lower than that of DeepDDI and DDIMDL. The reason is that the prediction result of CSMDDI-RESCAL relies heavily on the DDI network topology. If the prediction is between two new drugs, their embeddings from mapping function will include more errors, leading to the prediction result lower. In addition, note that the all the prediction results of task $S2$ is particularly lower than that of task $S1$. The main causes are that there are fewer new drugs and interactions in the test set, some DDI types even no interactions in scenario $S2$, result in the performance of AUC, AUPR and F1 score are very poor.

#### Visualization of the performance of each DDI type

**Fig. 5:**
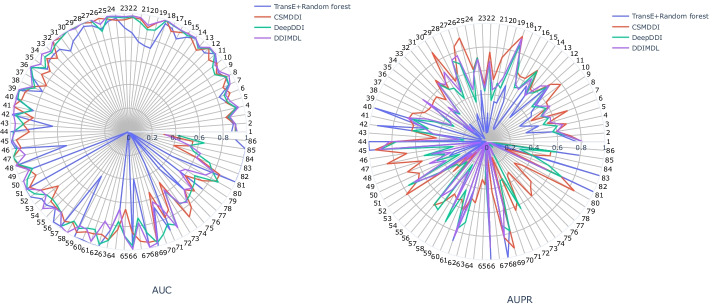
AUC and AUPR of all DDI types for different method

To further illustrate the effectiveness of CSMDDI, we also visualize the result of each type of DDIs in Fig. 5. There are 86 DDI types distributed around the circumference of the circle, and the serial numbers represent the different type of DDIs. It is ordered by its frequency occurred in the dataset, namely type 1 represents the highest frequency and type 86 represents the lowest frequency. From Fig. 5, we can find that in different models, the same DDI type prediction has slightly different, but the curve’s trends of different models are relatively consistent, especially in CSMDDI, DeepDDI and DMLDDI. CSMDDI also achieves a good performance in most types, especially in AUPR metric.

**Fig. 6:**
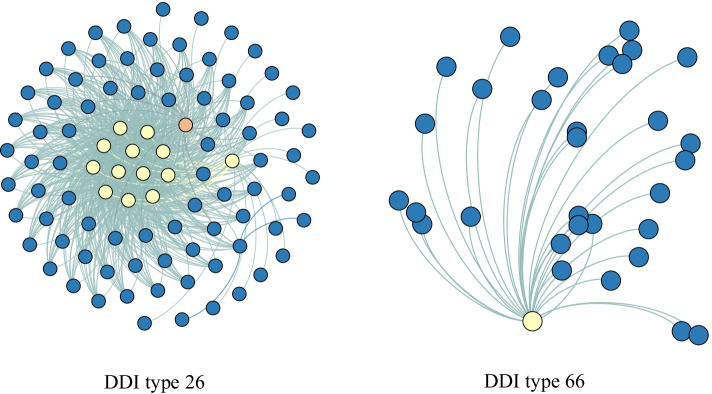
Subgraphs of DDI type 26 and type 66

From Fig. 5, we can also find that some DDI type are not consistent in the results among the comparison methods. For example, DDI type 26, the AUPR of CSMDDI is very high, while that of DeepDDI is very low; DDI type 66, the AUPR of CSMDDI is very low, while that of DeepDDI is very high. In order to analyze the causes, we draw the subgraphs of DDI type 26 and 66, as shown in Fig. 6. After statistical analysis, DDI type 26 has 94 drug nodes with an average degree of 16.7, while DDI type 66 has 32 drug nodes with an average degree of 1.9 and its topology is a star structure. Generally, a network with relatively dense edges is helpful to the training and easier to predict. This is because the embedding model can capture the network structure characteristics easily. While for a star structure network such as DDI type 66, it is not easy to learn and predict. The reason is that the average degree in this network type is too small and leading to the embedding learning is insufficient. It also is a puzzle hard to tackle in the research field of link prediction.

## Conclusion

Drug safety is one of the hottest topics in daily medical practice, especially in approving new drugs or questioning the possibility of withdrawing drugs from the market. In order to predict DDIs effectively, we propose a CSMDDI model for single-type and multi-type DDI prediction in cold start scenarios. In CSMDDI, we use the RESCAL embedding method to obtain the embedding representations of the drugs and DDI types. And then learning a mapping function between network topology and drug’s attribute, which can bridge new drugs attribute to their embeddings. This step is the core to handle the cold start scenario. By comparing other embedding methods and DeepDDI and DDIMDL, CSMDDI achieve a good performance both in single-type and multi-type DDI predictions in the cold start scenarios. In the future, more network embedding models will be tested in CSMDDI, the model with the best performance might be found under different conditions (data sets of different sizes, prediction tasks of different targets), to meet different needs in the real world. In addition, some nonlinear mapping methods can also be integrated in the second step of our framework.

## Supplementary Information

**Additional file 1.** This file contains a list of the 86 pharmacological types of DDI we have classified.

## Data Availability

The dataset and source code can be accessed at https://github.com/itsosy/csmddi.

## Abbreviations

CSMDDI: A cold start multi-type drug–drug interaction prediction model
DDI: Drug–drug interaction
SVD: Singular value decomposition
GAE: Graph AutoEncoder
ADRs: Adverse drug reactions
DPIs: Drug–protein interactions
CTET: The binding proteins of each drug, including carriers, transporters, enzymes and targets
CV: Cross-validation
AUC: Area under the receiver operating characteristic curve
AUPR: Area under precision–recall curve
