# Supplementary material for: Predict multi-type drug–drug interactions in cold start scenario
Source: BMC Bioinformatics. 2022 Feb 16;23:75. doi: 10.1186/s12859-022-04610-4 (PMC8851772; doi:10.1186/s12859-022-04610-4)
Supplement: Supplementary file 1 — Additional file 1. This file contains a list of the 86 pharmacological types of DDI we have classified. [file 12859_2022_4610_MOESM1_ESM.pdf]

Supplementary Material

The 86 pharmacological types of DDIs

No.	Name of DDI types	Total Num
1	The risk or severity of adverse effects increase	53750
2	The metabolism decrease	36789
3	The serum concentration increase	26297
4	the hypotensive activities increase	11076
5	The serum concentration decrease	10963
6	The therapeutic efficacy decrease	8044
7	the QTc-prolonging activities increase	6282
8	The metabolism increase	5250
9	the central nervous system depressant (CNS depressant) activities increase	4908
10	the antihypertensive activities decrease	3219
11	the anticoagulant activities increase	3006
12	the vasoconstricting activities decrease	2254
13	The therapeutic efficacy increase	2240
14	the hypoglycemic activities increase	2211
15	the antihypertensive activities increase	2086
16	The risk or severity of hypotension increase	1834
17	the bradycardic activities increase	1419
18	the hypokalemic activities increase	1322
19	the neuroexcitatory activities increase	1255
20	the excretion rate which could result in a higher serum level decrease	1096
21	the hypertensive and vasoconstricting activities increase	1002
22	the sedative activities increase	936
23	the serotonergic activities increase	932
24	the antiplatelet activities increase	846
25	the atrioventricular blocking (AV block) activities increase	795
26	the thrombogenic activities of Ethinyl increase	784
27	the cardiotoxic activities decrease	739
28	the bronchodilatory activities decrease	541
29	the tachycardic activities increase	469
30	the fluid increase	444
31	the stimulatory activities decrease	384
32	the sedative activities decrease	382
33	the nephrotoxic activities increase	366
34	the anticholinergic activities increase	339
35	the hyperkalemic activities increase	313
36	the anticoagulant activities decrease	306
37	the immunosuppressive activities increase	291

The 86 pharmacological types of DDIs

No.	Name of DDI types	Total Num
38	the arrhythmogenic activities increase	284
39	the neuromuscular blocking activities increase	266
40	The bioavailability decrease	230
41	the analgesic activities increase	218
42	The risk or severity of bleeding increase	200
43	the vasoconstricting activities increase	172
44	the constipating activities increase	149
45	effectiveness decrease	142
46	the cardiotoxic activities increase	138
47	the hyponatremic activities increase	130
48	the neuromuscular blocking activities decrease	125
49	the respiratory depressant activities increase	110
50	the vasopressor activities increase	108
51	the antipsychotic activities increase	99
52	the hypocalcemic activities increase	88
53	The risk or severity of rhabdomyolysis , myoglobinuria , and elevated creatine kinase (CPK) increase	84
54	the analgesic activities decrease	82
55	The risk or severity of myelosuppression increase	66
56	The risk or severity of hyperkalemia increase	64
57	the vasodilatory activities of Pentaerythritol_Tetranitrate increase	63
58	The risk or severity of edema formation increase	62
59	The risk or severity of sedation and somnolence increase	48
60	The risk or severity of congestive heart failure and hypotension increase	45
61	the ulcerogenic activities_Chloride increase	45
62	The risk or severity of angioedema increase	43
63	The absorption decrease	36
64	the hepatotoxic activities increase	34
65	the stimulatory activities increase	34
66	the bronchoconstrictory activities increase	31
67	the hyperglycemic activities increase	28
68	The risk of a hypersensitivity reaction increase	27
69	The risk or severity of heart failure increase	27
70	The risk or severity of myopathy and rhabdomyolysis increase	22
71	The risk or severity of renal failure increase	21
72	the excretion rate which could result in a lower serum level and potentially a reduction in efficacy increase	21
73	the diuretic activities decrease	20
74	the neurotoxic activities increase	17
75	the hypercalcemic activities increase	16
76	The risk or severity of ventricular arrhythmias increase	16

The 86 pharmacological types of DDIs

No.	Name of DDI types	Total Num
77	The risk or severity of convulsion increase	15
78	the dermatologic adverse activities increase	15
79	The risk or severity of serotonin syndrome increase	13
80	The risk or severity of severe leukopenia increase	12
81	The risk or severity of hyponatremia increase	12
82	The bioavailability increase	12
83	The risk or severity of ototoxicity and nephrotoxicity increase	11
84	The risk or severity of hypokalemia increase	10
85	The protein binding decrease	9
86	the antiplatelet activities decrease	7
